# Coordinated human sleeping brainwaves map peripheral body glucose homeostasis

**DOI:** 10.1016/j.xcrm.2023.101100

**Published:** 2023-07-07

**Authors:** Raphael Vallat, Vyoma D. Shah, Matthew P. Walker

**Affiliations:** 1 Center for Human Sleep Science, Department of Psychology, University of California, Berkeley, Berkeley, CA 94720-1650, USA

**Keywords:** NREM sleep, glycemia, diabetes, slow oscillations, sleep spindles, insulin resistance, heart rate variability, autonomic nervous system

## Abstract

Insufficient sleep impairs glucose regulation, increasing the risk of diabetes. However, what it is about the human sleeping brain that regulates blood sugar remains unknown. In an examination of over 600 humans, we demonstrate that the coupling of non-rapid eye movement (NREM) sleep spindles and slow oscillations the night before is associated with improved next-day peripheral glucose control. We further show that this sleep-associated glucose pathway may influence glycemic status through altered insulin sensitivity, rather than through altered pancreatic beta cell function. Moreover, we replicate these associations in an independent dataset of over 1,900 adults. Of therapeutic significance, the coupling between slow oscillations and spindles was the most significant sleep predictor of next-day fasting glucose, even more so than traditional sleep markers, relevant to the possibility of an electroencephalogram (EEG) index of hyperglycemia. Taken together, these findings describe a sleeping-brain-body framework of optimal human glucose homeostasis, offering a potential prognostic sleep signature of glycemic control.

## Highlights

• Coupled NREM sleep brainwaves predict superior next-day glucose control in two cohorts
• This sleep-glucose association is partially mediated by autonomic activity
• Sleep may regulate glycemic status selectively via insulin sensitivity
• NREM brainwaves thus offer a glycemic biomarker and a potential therapeutic target

## Graphical abstract

**Figure:**
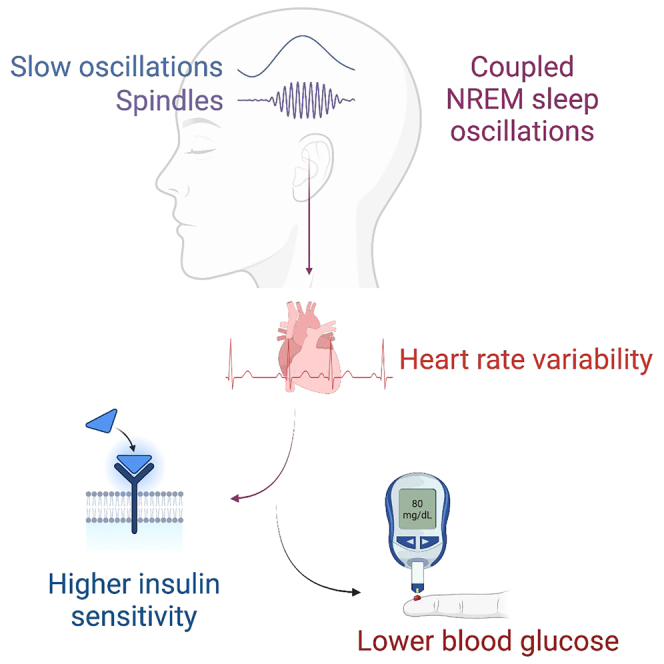

## Teaser

Vallat et al. demonstrate that the coupling of human slow oscillations and spindles during sleep predicts next-day insulin-dependent glucose regulation, suggesting a central sleep biomarker of metabolic homeostasis that supports peripheral glycemic equilibrium.

## Introduction

Diabetes—a condition of marked glucose dysregulation—is a major cause of death globally. The World Health Organization estimates that over 420 million people are suffering from the condition, which carries a direct societal cost of $760 billion each year.^1^ These preventable mortality and financial costs are projected to increase markedly over the next decade.^1^^,^^2^

Experimental studies in humans and animals have demonstrated that one causal factor impairing blood glucose equilibrium is insufficient sleep.^3^^,^^4^ Both acute and chronic partial sleep restriction, including that of non-rapid eye movement (NREM) slow-wave sleep, impair glucose tolerance and insulin sensitivity.^5^^,^^6^^,^^7^^,^^8^ Conversely, sleep extension improves glucose metabolism.^9^

But why? Currently, the mechanism(s) through which sleep optimally governs next-day glucose homeostasis in humans remains unknown. A recent seminal study in rodents has offered one candidate pathway.^10^ Specifically, hippocampal sharp-wave ripples—which are temporally coupled with NREM slow oscillations (SOs) and sleep spindles^11^^,^^12^^,^^13^—were associated with the moment-to-moment, top-down regulation of peripheral blood glucose through activation of the hypothalamus (which itself provides autonomic control of peripheral circulating hormones, including insulin).^10^^,^^14^

Collectively, these findings lead to the untested hypothesis that one function of synchronized (i.e., temporally coupled) NREM SO-sleep spindle events in humans is the brain-body regulation of optimal glucose homeostasis. More specifically, that both the extent and quality of coupled NREM SO-spindle events in humans would predict optimal next-day regulation of peripheral blood glucose levels.

## Results

In short (see STAR Methods and Table S1), a total of 647 humans with overnight polysomnography data and next-morning glucose and insulin measurements were analyzed to test the experimental hypothesis. Together with electrophysiological analysis of sleep oscillations and circulating morning measures of glucose, insulin resistance and pancreatic beta cell function were further quantified using the validated homeostatic model assessment of insulin resistance and beta-cell function (HOMA-IR and HOMA-B respectively; see STAR Methods and Figure S1 for details). Using these evaluations, we specifically tested the prediction that coupled NREM SO spindles the night before are associated with improved next-day peripheral blood glucose levels. To examine the robustness of these findings, we then tested these same associations between NREM SO-spindle coupling and peripheral blood glucose levels in an independent, larger replication cohort of 1,996 humans with the same sleep and glucose indices.

**Figure 1:**
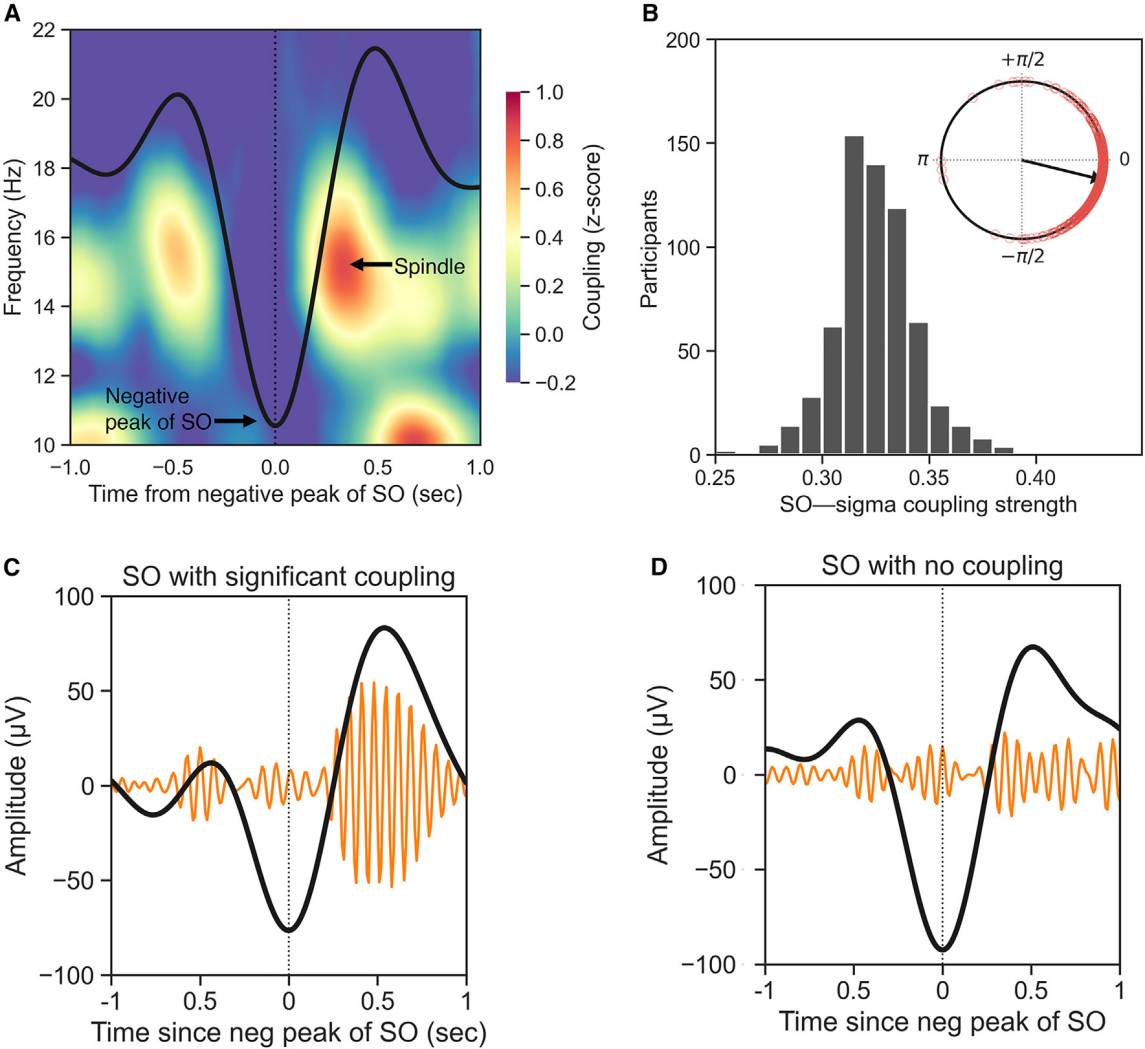
Slow oscillations are functionally coupled with sleep spindles (A) In human NREM sleep, slow oscillations (SOs; <1 Hz) are functionally coupled with sleep spindles, such that the phase of the SO modulates the amplitude of the spindle-related frequency band (12–16 Hz). The plot shows the average peak-locked SO calculated across all the participants (black thick line) and the associated time-frequency representation of the coupling strength.^15^ Warmer color indicates higher phase-amplitude coupling. The strongest coupling between SO and spindle-related activity occurs ∼0.4 s after the negative peak of the SO. (B) Histogram of the average SO-spindle coupling strength across all participants. The coupling strength is calculated using the normalized direct phase amplitude coupling (ndPAC) method.^16^ The circular plot shows the histogram of the preferred phase of the coupling. For most individuals, the maximum coupling occurs near the up phase of the SO (0°). (C) Example of a coupled SO. The thick black line shows the SO-filtered signal (0.3–1.5 Hz), whereas the orange lines show the associated spindle-filtered (12–16 Hz) signal, scaled by a factor of 4 for illustrative purposes. (D) Example of an uncoupled SO from the same individual as in (C). No statistical SO-spindle coupling was detected for this SO (see STAR Methods).

Focusing first on the cohort of 647 participants, and as expected, NREM SOs (<1 Hz) were functionally coupled with sleep spindles (mean, 87.6%; SD, 3.35; Table S1), such that the phase of the SO modulated the amplitude of the spindle-related frequency band (12–16 Hz), hereafter referred to as SO-spindle coupling (for conciseness). The strongest coupling between SO and spindle-related activity occurred ∼0.4 s after the negative peak of the SO (Figure 1A; Table S1). For most individuals, the maximum coupling occurred near the up phase of the SO (−12.15° ± 28.32°; Figure 1B; Table S1).

**Figure 2:**
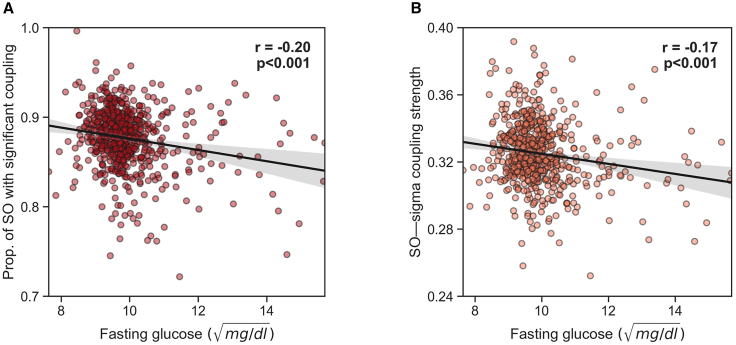
SO-spindle coupling predicts lower next-day fasting glucose in the CFS dataset (A) Partial correlation adjusted for age between the extent of SO-spindle coupling (i.e., the proportion of SOs that are significantly coupled, see STAR Methods) and next-day fasting blood glucose levels. (B) Partial correlation adjusted for age between SO-spindle coupling strength and next-day fasting blood glucose levels. Translucent bars represent 95% bootstrapped confidence intervals. Fasting glucose levels were normalized using a square-root transformation (see STAR Methods). Of note, both coupling measures remained significantly correlated with fasting glucose levels when removing fasting glucose values above 12 (= 144 mg/dL; r = −0.20, p < 0.001 and r = −0.15, p < 0.001, respectively).

Next, we tested the prediction that the degree of such coupling of NREM sleep oscillations was associated with glycemic control the following day. Supporting the hypothesis, greater SO-spindle coupling at night predicted lower next-day fasting blood glucose levels (partial correlation adjusted for age, r = −0.20, n = 631, p < 0.001; Figure 2A). Beyond the simple quantity of synchronized SO-spindle events, the strength of the temporal synchrony (meaning the precision of the timing of the coupling) between SOs and spindle activity was similarly associated with lower subsequent fasting blood glucose levels (partial r = −0.17, n = 631, p < 0.001; Figure 2B).

To date, multiple other factors have been identified that influence glycemic control beyond sleep. *Prima facie* examples include age, gender, race, body mass index (BMI), hypertension, and even certain sleep features, such as apnea-hypopnea index (AHI), the quantity of sleep, and specific sleep stages.^17^^,^^18^

To ensure that the relationship between SO-spindle coupling and blood glucose levels was robust, multilevel regression models were fitted to adjust for these known co-risk factors. With all factors included in the analysis model (age, gender, race, BMI, hypertension, AHI, sleep duration, sleep efficiency, and family as a random effect), the relationships between higher SO-spindle coupling and lower next-day fasting blood glucose levels remained significant (p = 0.001 and p = 0.020 respectively; Tables S2 and S3). This suggests a statistically independent contribution of coordinated sleep oscillations to the mapping of next-day blood glucose control beyond these other classic factors known to govern glycemic state.

An additional sensitivity analysis was conducted adjusting for diabetes status as an additional covariate, to check if this association is different in normoglycemic versus diabetic individuals. The association between coupling quantity and lower fasting blood glucose levels remained at trending significance when including diabetes status as an additional covariate in the regression analysis (β = −1.79, p = 0.063). However, the association between coupling strength and fasting glucose did not remain significant (β = −2.59, p = 0.133).

Two other important risk factors for metabolic health are smoking status and education level.^19^^,^^20^ These two factors were not included in the main regression model because of a high missingness of data, which resulted in the exclusion of ∼30% of participants from the analysis. However, the association between coupling quantity and lower fasting blood glucose levels remained significant when including smoking status and education level as additional covariates in the regression analysis (β = −4.22, p = 0.002). This was not true of the coupling strength (β = −2.65, p = 0.29). Based on *in vivo* cellular recordings in animal models, the proportion of SO-spindle coupling may be a better metric of hippocampal sharp-wave ripple density, which is causally associated with peripheral blood glucose levels via a hypothalamic signaling pathway,^10^ than the strength of that brainwave coupling.^13^ Taken together, such selectivity suggests that the proportion of coupled SO-spindle events represents the most sensitive sleep biomarker of human next-day glucose homeostasis.

Beyond the predictive relationships with fasted blood glucose levels, similar associations were observed with 2-h postprandial glucose values following an oral glucose tolerance test (OGTT). To assess whether overnight sleep was associated with next-day OGTT glucose levels, we re-ran the models reported above for fasting blood glucose and SO-spindle coupling, similarly adjusted for known risk factors including age, gender, race, BMI, hypertension status, AHI, sleep duration, and efficiency. Here again, both the proportion (β = −5.36, p = 0.037) and strength (β = −9.31, p = 0.044) of SO-spindle coupling were significantly associated with lower (superior) next-day OGTT values. Therefore, SO-spindle coupling demonstrated predictive relationships with glycemic status in both the fasted state, and the body’s dynamic reaction to a metabolic glucose challenge that requires a functional regulatory glycemic-control response.

**Figure 3:**
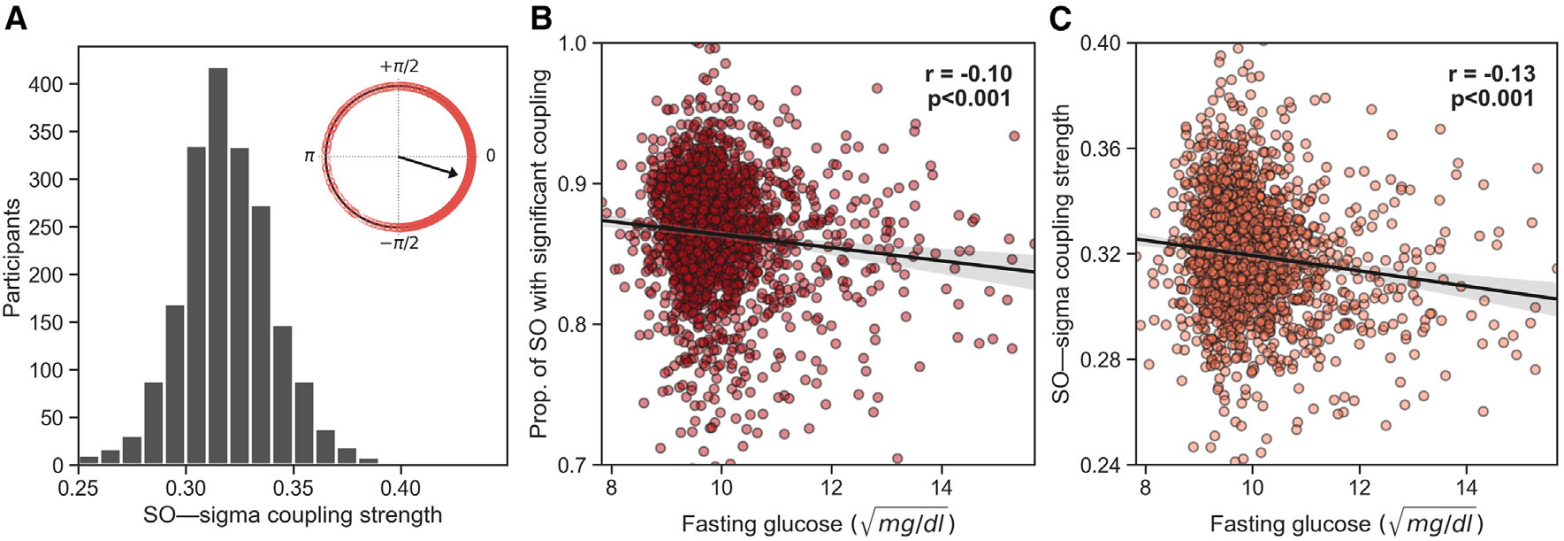
SO-spindle coupling during sleep is a prominent marker of glucose homeostasis, in an independent (MESA) dataset (A) Histogram of the average SO-spindle coupling strength across all participants in the MESA dataset. The coupling strength is calculated using the ndPAC method.^16^ The circular plot shows the histogram of the preferred phase of the coupling. For most individuals, the maximum coupling occurs near the up-phase of the SO (0°). (B) Partial correlation adjusted for age between the extent of SO-spindle coupling (i.e., the proportion of SOs that are significantly coupled, see STAR Methods) and next-day fasting blood glucose levels in the MESA dataset. (C) Partial correlation adjusted for age between SO-spindle coupling strength and next-day fasting blood glucose levels. Translucent bars represent 95% bootstrapped confidence intervals, in the MESA dataset. Fasting glucose levels were normalized using a square-root transformation (see STAR Methods).

The next series of analyses sought to test the replicability and robustness of the SO-spindle coupling reflecting a marker of glucose homeostasis in a larger, independent cohort. For this purpose, we examined the Multi-Ethnic Study of Atherosclerosis (MESA^21^; see STAR Methods; Figure 3A; Table S4) of over 1,900 participants involving fasted glucose measurements and overnight polysomnography sleep recording.

Consistent with the results in the first independent cohort, SO-spindle coupling during NREM sleep once again predicted superior fasting peripheral blood glucose in this second cohort (partial correlation adjusted for age, r = −0.103, n = 1968, p < 0.001; Figure 3B). Moreover, the strength of the temporal synchrony between SO-spindle coupling was similarly associated with lower fasting blood glucose levels, as in the first cohort (partial r = −0.130, n = 1968, p < 0.001 respectively; Figure 3C).

In addition, both the proportion and strength of SO-spindle coupling remained significantly associated with fasting blood glucose after adjusting for assessed risk factors (age, gender, race, BMI, hypertension, AHI, and the quantity and quality of sleep; p = 0.034 and p = 0.011, respectively; Tables S5 and S6).

Taken together, these results replicate the association between SO-spindle coupling and fasting blood glucose levels from the first cohort dataset and support the association of SO-spindle coupling as a central brain marker of peripheral body glycemic status.

Glucose homeostasis is governed by several independent mechanisms, key among them being the function ability of pancreatic beta cells. Pancreatic beta cells initially sense increases in glucose and lead to the release of insulin and, separately, the sensitivity of cells in the body to the signal of insulin (the impairment of which can result in insulin resistance). Having established the association between coupled NREM sleep oscillations and peripheral body glucose state, we next sought to determine whether this sleep biomarker was mapping one or both of these glucose homeostasis pathways within the first main cohort. This was accomplished using the added measures of HOMA-IR, which provides a representation of insulin resistance/sensitivity, while HOMA-B provides an index of insulin secretory function.^22^^,^^23^^,^^24^

**Figure 4:**
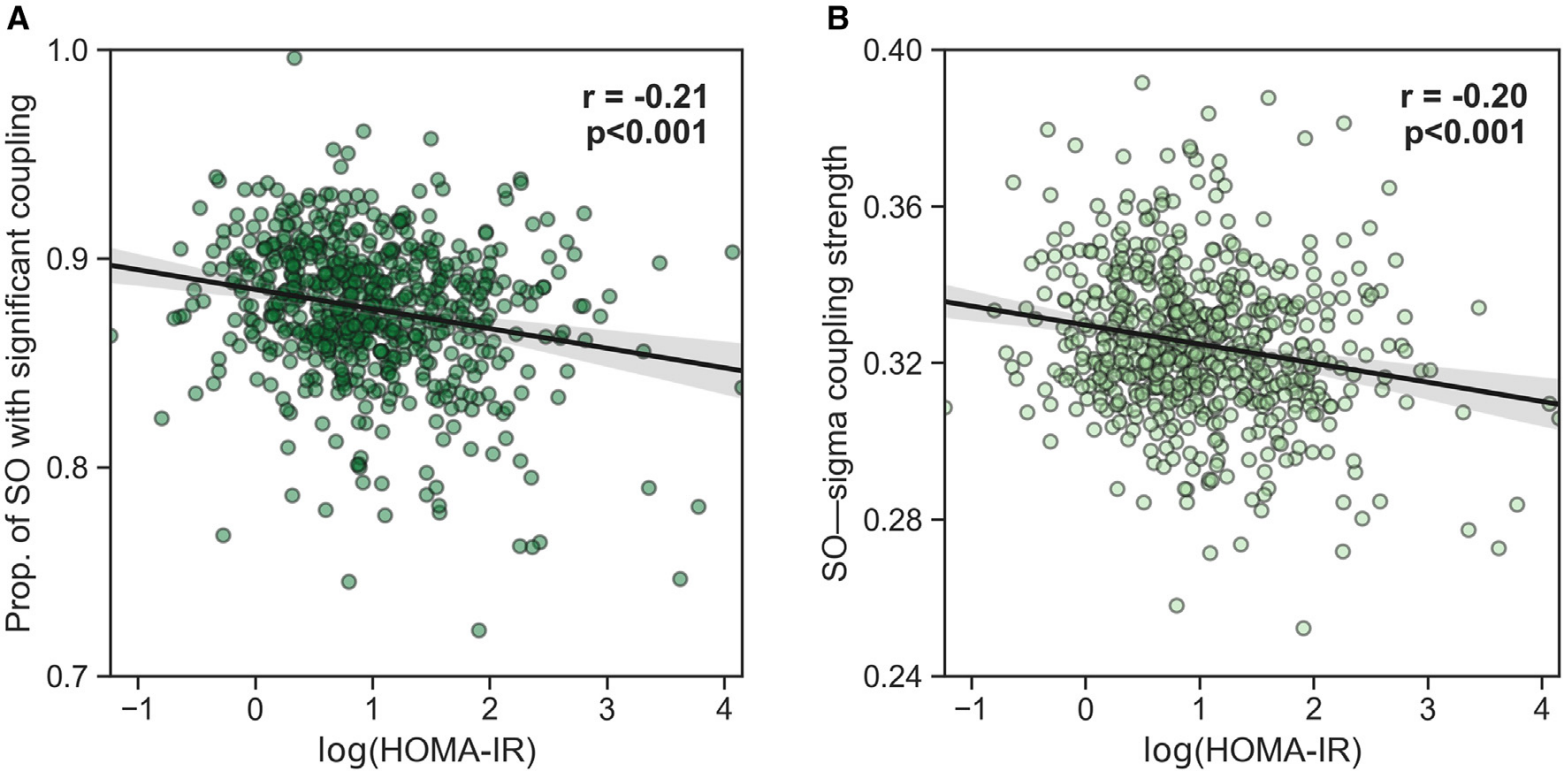
Insulin resistance is significantly correlated with the coupling between SOs and spindle-related activity in the CFS dataset (A) Partial correlation adjusted for age between the extent of SO-spindle coupling (i.e., the proportion of SOs that are significantly coupled, see STAR Methods) and next-day HOMA-IR. (B) Partial correlation adjusted for age between SO-spindle coupling strength and next-day HOMA-IR. Translucent bars represent 95% bootstrapped confidence intervals.

Lower SO-spindle coupling predicted higher (i.e., worse) next-day insulin resistance the following day, quantified using the validated metric of HOMA-IR—the marker of insulin sensitivity (r = −0.213, n = 634, p < 0.001; Figure 4A). However, suggesting a mechanistic dissociation, no such sleep associations were identified with HOMA-B, reflecting pancreatic beta cell insulin secretion (r = −0.072, n = 626, p = 0.074).

Furthermore, both the proportion and strength of SO-spindle coupling remained significantly associated with HOMA-IR after adjusting for all aforementioned risk factors (p = 0.005 and p = 0.016, respectively; Tables S7 and S8).

Beyond the simple number (quantity) of synchronized SO-spindle events, the quality of coupling (indexed by the strength of temporal synchrony between SOs and spindle activity) was similarly associated with improved next-day blood glucose homeostasis, as assessed by fasted glucose levels (r = −0.170, n = 634, p < 0.001; Figure 2B) as well as next-day insulin sensitivity as measured by HOMA-IR (r = −0.197, n = 634, p < 0.001; Figure 4B). Once again, there was no such association with the pancreatic beta cell secretion function using the measure of HOMA-B (r = −0.066, n = 626, p = 0.097).

Such results further support the proposal that the association between NREM sleep oscillations and next-day glucose homeostasis is best understood through altered insulin sensitivity within the body rather than changes in insulin secretion by way of pancreatic beta cell function.

One candidate pathway explaining the association between SO-spindle coupling and next-day glucose homeostasis is an alteration in heart rate variability (HRV) during sleep, an indirect measure of autonomic parasympathetic activity. Accordingly, we conducted a mediation analysis which revealed that HRV (see STAR Methods) significantly mediated the association between both proportion and strength of SO-spindle coupling and next-day fasting glucose levels in the MESA dataset (indirect effect, p = 0.0014 and p = 0.0008 respectively; Figures S2A and S2B). Specifically, the greater the proportion of coupled SOs during sleep, the higher the HRV (indicative of greater parasympathetic dominance) during sleep (p = 0.001; adjusted for all aforementioned cofactors), which, in turn, was linked to superior (i.e., lower) next-day fasting blood glucose levels (p < 0.001). In the Cleveland Family Study (CFS) dataset, a similar effect was observed for HRV during sleep mediating the association between the proportion of SO-spindle coupling and insulin resistance, with trending significance (indirect effect, p = 0.076; Figure S4A), such that a higher proportion of coupled SO-spindle events was associated with higher HRV (p = 0.037) through the statistical mediation pathway, further predictive of lower insulin resistance (p = 0.022). However, HRV in the CFS cohort did not significantly mediate the association between the proportion (indirect effect, p = 0.135; Figure S3A) or strength (indirect effect, p = 0.66; Figure S3B) of SO-spindle coupling and next-day fasting glucose levels.

**Figure 5:**
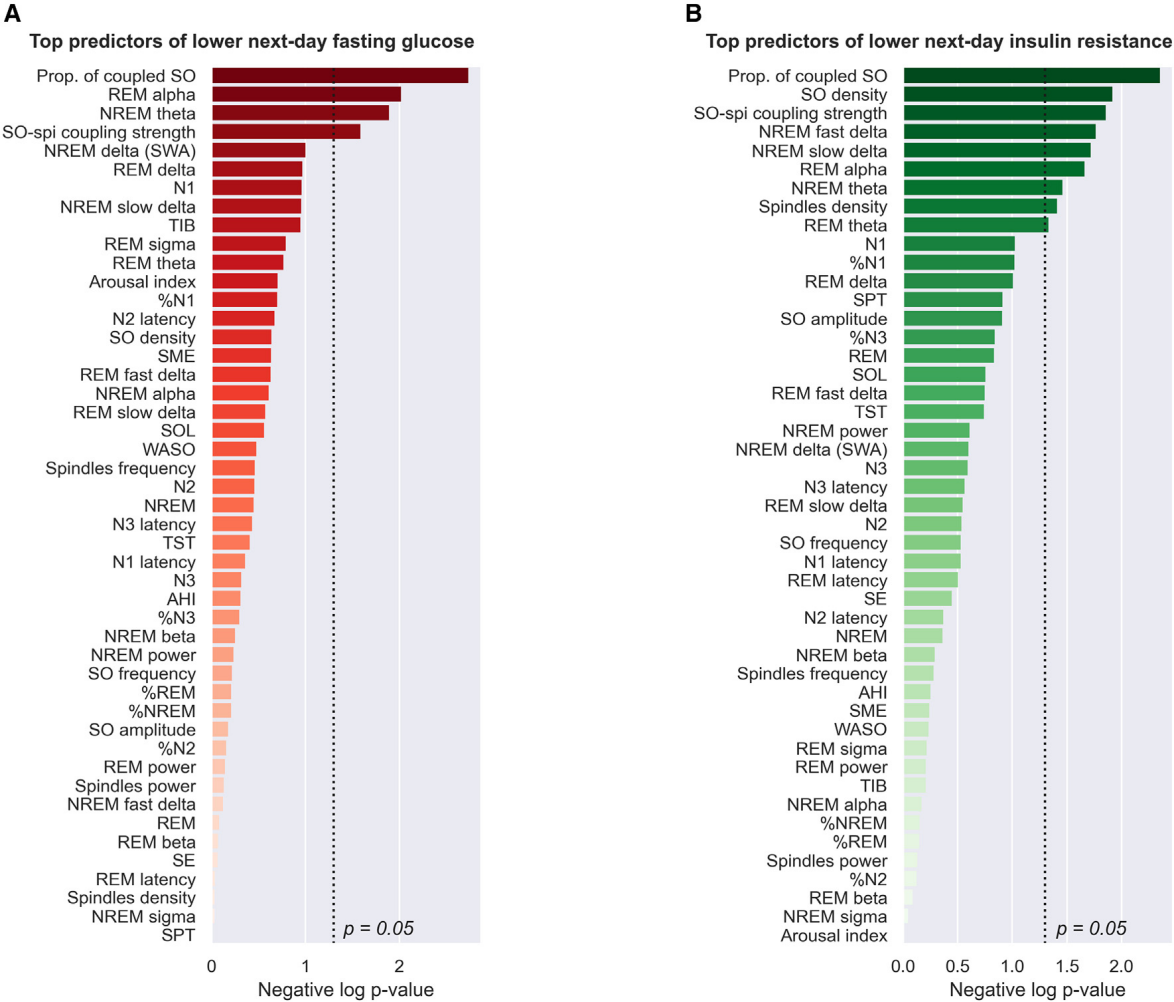
SO-spindle coupling is the top sleep predictor of next-day glucose homeostasis (A) Top sleep predictors of lower next-day fasting glucose, ranked in descending order of significance (negative log10 p value). (B) Top sleep predictors of lower next-day insulin resistance (HOMA-IR) ranked in descending order. The proportion of SOs with significant coupling was the best sleep predictor of both fasting glucose and insulin resistance. Unadjusted two-tailed p values were obtained by fitting, for each sleep predictor separately, a multilevel regression model adjusted for age, gender, BMI, race/ethnicity, hypertension, and family identification. A total of 47 sleep parameters were included in the rank analysis. NREM refers to N2 + N3 sleep (N1 excluded). A full description of these parameters is provided in Tables S9 and S10.

Since impaired glucose function has been associated with broad, macro-level sleep features such as sleep apnea severity, sleep duration, and certain stages of sleep,^17^^,^^18^ we next examined the predictive sensitivity of our *a priori* micro-sleep measures of SO-spindle oscillation coupling and how it ranked relative to all other sleep metrics. Notably, after adjusting for known risk factors for glucose homeostasis (specifically age, gender, BMI, hypertension, and family as a random effect), SO-spindle coupling was the single strongest sleep predictor of next-day fasting glucose levels and insulin resistance relative to all other traditional sleep metrics (Figure 5). This included the amount of time (number of minutes and percentage) in each sleep stage (N1, N2, N3, and rapid eye movement [REM]), sleep duration and sleep efficiency, wake after sleep onset (WASO), the arousal index, sleep apnea severity as measured with the AHI, individual morphological features of either SOs or spindles (density, frequency, amplitude), and spectral band power in REM or NREM sleep (slow delta, fast delta, total delta, theta, alpha, sigma, beta; see Figure 5; STAR Methods). Together, these findings indicate that SO-spindle coupling is a predominant and prominent marker of next-day glucose homeostasis.

For purely ecological relevance, we examined the effect-size association between superior to inferior SO-spindle coupling and next-day glucose homeostasis balance. Going from the first percentile of the proportion of coupled SOs (77%) to the 99^th^ percentile (94%) represented a decrease of 13.2 mg/dL in fasting blood glucose levels (holding all other covariates constant). Similarly, going from the first percentile to the 99^th^ percentile value of the coupling strength represented a decrease of 9.9 mg/dL in fasting glucose levels. For reference, the current Centers for Disease Control and Prevention (CDC) guidelines indicate that a difference of ∼15 mg/dL in fasting glucose levels reflects the difference between an individual in a normoglycemic zone to being prediabetic (e.g., from 95 to 110 mg/dL) or from a prediabetic state to being diabetic (e.g., from 115 to 130 mg/dL).

## Discussion

Taken together, these findings support a NREM sleep-oscillation brain-body framework of glucose homeostasis in humans, one that describes a mapped association between prior SO-spindle coupling and next-day glucose homeostasis.

Prior observations in rodents have demonstrated that hippocampal ripples during sleep decrease peripheral blood glucose levels moment-to-moment, in part through a hypothalamic signaling pathway.^10^ Considering that coupled SO-spindle activity coincides subcortically with hippocampal sharp-wave ripples,^11^^,^^13^ our results indicate the presence of a similar sleeping-brain—glycemic association observable in humans. Furthermore, the report in rodents reported that isolated ripples did not show an association with peripheral glucose levels, whereas bursts of hippocampal ripples did. Independent of glucose control, there is also evidence in humans that the nesting of ripples in spindle troughs during SO-spindle coupling is more strongly associated with ripple bursts, compared with isolated ripples.^11^^,^^12^^,^^13^^,^^25^ These findings indicate the possibility that the SO-spindle coupling-glucose homeostasis association we identify here is driven by collective burst trains of hippocampal sharp-wave ripples, as opposed to isolated ripples. Such oscillation trains may therefore underlie part of the mechanistic pathway accounting for the associations reported here in humans.

It is also important to note, however, that the above NREM sleep-oscillation framework of brain-to-body glucose homeostasis can be considered across at least two different timescales, which may not be mutually exclusive. The first, as we describe here in humans, involves a temporally longer (hours), feedforward association, such that NREM SO-spindle coupling predicts superior next-day glucose homeostasis. The second, previously observed in rodents,^10^ involves a short-term feedback loop (seconds to minutes) between hippocampal sharp-wave ripple activity and concurrent changes in circulating glucose during sleep. Both processes, either independently or interactively (e.g., moment-to-moment changes in glucose across the night cumulatively determine next-morning glucose status), may aid in generalized glycemic homeostasis. Importantly, these two pathways offer disease insights into the brain (sleep)-body (glucose) mechanisms that help explain the well-characterized associations between short and disrupted sleep, hyperglycemia, and type-II diabetes.^3^

Moreover, and critical from a clinical and public-health perspective, our findings demonstrate that these associations remained significant when controlling for prototypical factors that themselves are known to affect blood glucose, including age, gender, race, BMI, hypertension, and even sleep measures, such as the AHI, and the quantity and quality of sleep. That is, an association between SO-spindle coupling and glucose homeostasis that is independent of other cofactors influencing glycemic control, both in measures of fasted blood glucose assessment and following the standard metabolic challenge of an OGTT.

Importantly, this association between SO-spindle coupling and peripheral glucose homeostasis was also validated in an independent, larger replication dataset, suggesting that the effects are less likely to be driven by single cohort-specific idiosyncrasies (though additional cohort replications are required). Nevertheless, the replication of the association between SO-spindle coupling and fasting blood glucose levels at least offers some appended support to the framework of SO-spindle coupling metric in a predictive or supervisory role of glucose homeostasis,^10^ above and beyond other traditional sleep metrics.

Adding to these insights, SO-spindle coupling predicted next-day improved (enhanced) insulin sensitivity, but not pancreatic beta cell function. That is, a potential dissociation between two key glycemic control mechanisms: (1) the ability of pancreatic beta cells to respond to the glucose status of the body, which can release insulin in the presence of sensed high glycemic load; and (2) the sensitivity of cells within the body to that consequential signal of insulin released by the pancreas, resulting in the cellular uptake of glucose from the blood.^17^ Our findings suggest that the link identified between SO-spindle coupling and glucose homeostasis is not one associated with a dual-action regulation of glycemic control. Rather, relationships were observed only for the measure of HOMA-IR (indexing insulin sensitivity within the body), and not HOMA-B, reflecting pancreatic beta cell sensing and the release of insulin. Therefore, the association with blood glucose stasis appears to be most parsimoniously explained by a link between NREM sleep oscillations and a select alteration in insulin sensitivity,^26^^,^^27^ rather than regulating pancreatic beta cell function or insulin synthesis/secretion.^24^^,^^28^

The identified alterations in fasting glucose levels and impairments in the oral glucose tolerance test (OGTT) each reflect different aspects of insulin resistance. The former measured in the fasted state has been linked to hepatic insulin resistance, while the latter OGTT response is primarily associated with impaired muscle insulin resistance.^29^ It is important to note, however, that the OGTT findings were only assessed in normoglycemic individuals. Future examinations in hyperglycemic cohorts are needed to explore whether this sleep-associated allostatic response (i.e., OGTT) is different in diabetes.

One possible mechanism explaining the recognized link between deficient sleep and impaired blood glucose control is an alteration of autonomic sympathovagal balance resulting in a biased state of sympathetic activity over parasympathetic activity,^30^ which may chronically lead to insulin resistance and metabolic dysfunction.^31^ Addressing this question, we conducted a mediation analysis to test whether coupled NREM oscillations and superior glycemic status were mediated through an association with increased parasympathetic autonomic activity during sleep. Heart rate variability (HRV), an indirect measure of autonomic parasympathetic activity, significantly mediated the association between both the proportion of SO-spindle coupling and next-day fasting glucose levels in the MESA dataset, though it should be noted that this relationship did not reach significance within the CFS cohort dataset. Based on this, parasympathetic activity may only be one partial pathway linking SO-spindle coupling with next-day glucose homeostasis. Other such pathways may exist that account for the additional variance in mediation that is not explained by parasympathetic activity in this sleep-glycemic relationship.

To date, associations between sleep loss, blood glucose status, and diabetes risk have productively focused on traditional sleep statistics, including sleep duration, sleep efficiency, amount of each sleep stage (particularly the loss of deep NREM sleep^7^^,^^8^), and markers of sleep disorders (e.g., AHI).^3^^,^^32^ However, exactly what it is within sleep that accurately maps glycemic control in humans has remained unknown. Addressing this issue, we demonstrate that SO-spindle coupling is not only a sensitive glycemic index but, of all sleep features, including sleep stages, and all other sleep electrical oscillation spectra, such coupling offers the highest predictive sensitivity of next-day glucose homeostasis. Indeed, this predictive relationship with glucose status exceeded that of all other sleep measures assessed, including total sleep amount, sleep efficiency, NREM slow-wave sleep, as well as sleep apnea severity (AHI score). Our findings in no way challenge these now robust links between those aforementioned sleep measures and diabetes risk and/or blood glucose status.^3^^,^^33^ Rather, our results establish the measure of SO-spindle coupling as an additional, independent contributing feature of sleep, one that offers insights into potential disease pathways associated with diabetes considering recent rodent data causally linking SO-spindle coupling with momentary glucose regulation.

In conclusion, our findings suggest a sleeping-brain—glycemic-body framework of insulin-associated glucose homeostasis in humans, and further re-emphasize the importance of sleep in the clinical management of hyperglycemia and diabetes.

### Limitations of the study

Our study must be appreciated within the context of important limitations. First, although our findings describe a temporal association between sleep the night before and peripheral glucose homeostasis, the results do not establish causality. The mechanism(s) by which SO-spindle coupling affects next-day glucose homeostasis in humans needs further exploration. Given that our data are non-invasive and only measure next-day glucose, we are unable to gain causal and temporal insight into the association between hippocampal sharp-wave ripple activity and SO-spindle coupling and glucose homeostasis. Future studies in intracranial patients, along with continuous glucose monitoring, would help provide further mechanistic insight. However, multiple studies have shown that hippocampal sharp-wave ripples are temporally coupled with NREM SOs and sleep spindles,^11^^,^^12^^,^^13^ making SO-spindle coupling a promising non-invasive marker of hippocampal sharp-wave ripple bursts. Our findings motivate the design of studies capable of testing bidirectional causality (e.g., manipulating SO-spindle coupling in humans^34^ to alter glucose regulation or vice versa). Second, the effect sizes observed in this study are, as anticipated, in the small-to-moderate range, and similar to those recently reported in rodents.^10^ This is expected, considering that an individual’s blood glucose level is determined by multiple factors, including genetics, food intake/diet, and gut microbiome.^35^^,^^36^^,^^37^ Sleep—an indirect lifestyle factor—is therefore anticipated to account for a somewhat modest, yet still clinically meaningful, proportion of between-person variability in glucose levels.^38^ This was affirmed in our findings by the significant difference between those in the upper and lower quartiles of SO-spindle coupling activity. Future studies that provide longitudinal repeated assessment will help examine how potential individual differences in baseline general health could contribute to differences in SO-spindle coupling and metabolic deficiencies. Finally, measures of glucose in the main dataset (CFS) were assessed in the morning for closest proximity to sleep, affording a test of the sleep-dependent hypothesis. Nevertheless, these measures do not provide insight into glucose regulation across the entire day, although it should be noted that there is a significant correlation between blood glucose levels measured across the day.^39^ Still, temporal knowledge of glycemic status across the day can have important benefits to understanding metabolic dysfunction, requiring continuous glucose monitoring across the 24-h period as an ideal next experimental step.^40^^,^^41^

## STAR★Methods

### Key resources table

**Table:** 

REAGENT or RESOURCE	SOURCE	IDENTIFIER
**Deposited data**		

Cleveland Family Study data	Zhang et al. 2018^42^; Redline et al. 1995^43^	https://sleepdata.org/datasets/cfs
Multi Ethnic Study of Atherosclerosis data	Zhang et al. 2018^42^; Chen et al. 2015^44^	https://biolincc.nhlbi.nih.gov/studies/mesa/

**Software and algorithms**		

YASA	Vallat and Walker 2021^45^	https://github.com/raphaelvallat/yasa
Tensorpac	Combrisson et al. 2020^46^	https://github.com/EtienneCmb/tensorpac
Pingouin	Vallat 2018^47^	https://pingouin-stats.org/
Code for all data preprocessing and analysis	This paper	https://github.com/raphaelvallat/vallat2023_coupling_glucose

### Resource availability

#### Lead contact

Further information and requests for resources and reagents should be directed to and will be fulfilled by the lead contact, Raphael Vallat (raphaelvallat9@gmail.com).

#### Materials availability

This study did not generate new materials.

### Experimental model and subject details

Two independent cohorts were used to test the hypothesis. The first (main cohort) was the Cleveland Family Study data set (CFS;^42^^,^^43^), and the second (replication cohort) was the Multi-Ethnic Study of Atherosclerosis (MESA;^21^) data set. Both the CFS and the MESA datasets followed the guidelines of the National Sleep Research Resource (NSRR), and Institutional Review Board (IRB) approval was obtained at each study site.

The former Cleveland Family Study (CFS) data set is a longitudinal family-based epidemiological study of sleep apnea with over 2400 participants. Families were selected based on the presence of a proband diagnosed with Obstructive Sleep Apnea (OSA;^48^). Neighboring families without a diagnosis of OSA were used as controls. A subset of 728 participants was selected for a study that involved collecting sleep, cardiovascular and metabolic measures, between July 2001 and June 2005 (visit 5). Prepubertal children were excluded from subsequent analyses by using 15 years old as the cut-off age (n = 73, 655 participants remaining). The protocol was approved by the institutional review boards of the local hospitals from where the participants were recruited. All participants provided informed written consent.

The latter Multi-Ethnic Study of Atherosclerosis (MESA^21^) data set is a multi-center, longitudinal investigation of factors associated with the development of cardiovascular disease. There have been five follow-up visits to date, approximately once every two years. All participants provided written informed consent and all MESA activities were approved by the institutional review boards of the participating institutions. All subsequent analyses are based on the MESA Exam 5, which was collected from 2010 to 2013.

### Method details

#### Measurement of glycemic levels, insulin sensitivity and health covariates

##### CFS

Fasting glucose and insulin values were derived from all individuals (n = 728) using blood samples collected via venipuncture at 7 A.M. on the morning after PSG.^48^ In non-diabetic participants (n = 596), this was followed by the administration of an oral glucose tolerance test (OGTT). During the oral glucose tolerance test, participants orally consumed 75 grams of anhydrous glucose, and glucose levels were measured 2 hour later via venipuncture. OGTT values were measured as 2 hour post glucose serum load (in mg/dl). Impaired glucose tolerance criteria were defined by self-reported use of diabetes medication, as fasting glucose ≥110 mg/dL, or as 2 hour post glucose serum load ≥140 mg/dL. A square root transformation was used to reduce skewness in fasting and postprandial glucose levels and thus minimize the influence of outliers, consistent with prior assessment measures.^37^

Insulin resistance and pancreatic beta cell function were quantified using the standardized homeostasis assessment model (HOMA-IR and HOMA-B respectively) scores. HOMA-IR was calculated as fasting serum insulin multiplied by fasting plasma glucose (in mg/dL), divided by 405, as described previously.^22^^,^^23^^,^^49^ HOMA-B was calculated as fasting serum insulin multiplied by 360, divided by fasting plasma glucose (in mg/dL) minus 63.^22^^,^^24^ HOMA-IR and HOMA-B values were then log transformed to reduce skewness, consistent with standard practices.^50^^,^^51^ High scores indicate low insulin sensitivity, or high insulin resistance.

Before coming in for their PSG session, all participants completed the Cleveland Health and Sleep Questionnaire, which is a standardized and validated questionnaire assessing sleep habits and symptoms, medical history, health habits, and medication use, including diabetic and antihypertensive medications. BMI was measured as the ratio of weight to the square of height (kg/m^2^). Weight was measured to the nearest 0.1 kg using a calibrated scale. Height was measured to the nearest centimeter using a wall-mounted stadiometer.

##### MESA

Fasting glucose was measured during the MESA Exam 5 clinic visit. Participants fasted for 12 hours and avoided smoking and heavy physical activity for 2 hours before the examination. Fasting blood samples were drawn between 7:30 A.M. and 10:30 A.M.. Fasting blood glucose (serum) was measured by the glucose oxidase method on the Vitros analyzer (Johnson & Johnson Clinical Diagnostics, Rochester, New York).^52^ As in CFS, fasting glucose values outside the range of 60–250 mg/dL were masked (n = 8). Then, a square root transformation was used to further reduce skewness in fasting glucose levels and minimize the influence of outliers, consistent with prior assessment measures.^37^ Age, gender, race/ethnicity, smoking status, education, and income were collected at MESA Exam 5 via self-report questionnaires.

#### EEG analysis

##### Sleep recording and sleep staging

###### CFS

Fourteen-channel overnight PSG recordings were collected using Compumedics E Series System, at a dedicated clinical research facility. Details about the montage and sampling rate can be found here. Sleep scoring was performed by trained research technologists, using R&K rules.^53^ For subsequent analyses, NREM stages 3 and 4 were collated into a single stage (N3) to conform with the most recent guidelines.^54^

###### MESA

Sleep studies were scheduled to occur after the MESA Exam 5 clinic visit. The average gap between the MESA sleep study and MESA Exam 5 clinic visit was 341 days, with a standard deviation of 200 days. At-home full overnight PSG recordings were collected in 2237 participants from the parent cohort (age range = 54–95 years) using the Compumedics Somte System (Compumedics Ltd., Abbotsford, Australia). The recording montage consisted of three cortical EEG (central C4-M1, occipital Oz-Cz, and frontal Fz-Cz leads), bilateral EOG, chin EMG, as well as several other sensors to measure heart rate, respiration and leg movements.

### Quantification and statistical analysis

#### Spectral analyses

EEG power in specific bands were calculated separately for NREM sleep (excluding N1) and REM sleep, using a Welch periodogram with a 4-second hamming window. Spectral bands were defined as: slow delta (0.5–1.25 Hz), fast delta (1.25–4 Hz), total delta (i.e. slow wave activity [SWA], 0.5–4 Hz), theta (4–8 Hz), alpha (8–12 Hz), sigma (12–16 Hz), beta (16–30 Hz). EEG powers were expressed as a proportion of the total power summed across all bands. The correlation analyses also included the total summed power in NREM and REM (expressed in microVolts-squared).

#### Slow oscillations event-locked phase-amplitude coupling

All EEG analyses for the CFS dataset were conducted on the C3-M2 channel, after downsampling to 100 Hz and inverting the polarity (to fix a known issue, see here). All EEG analyses for the MESA dataset were conducted on the C4-M1 channel. PSG data were sampled at 256 Hz and a hardware low-pass filter with a cutoff frequency of 100 Hz was applied during recording. Nocturnal recordings were transmitted to the centralized reading center at Brigham and Women’s Hospital and data were scored by trained technicians using current guidelines.

Slow oscillations (SO) detection was performed on NREM sleep (excluding N1 sleep) using the YASA Python library.^45^ The algorithm uses amplitude and duration thresholds^55^^,^^56^ to detect SO on the bandpass-filtered signal (0.3–1.5 Hz), coupled with an outlier removal step to remove invalid events. Based on previous findings showing that the standard amplitude threshold of 75 μV is not adequate for older adults,^57^ a more liberal amplitude threshold of 60 μV for peak-to-peak amplitude and 32 μV for the negative peak amplitude was chosen. For each PSG night, the average SO density (= number of SO per min of NREM), frequency (Hz) and amplitude (μV) were calculated.

To calculate event-locked cross-frequency coupling,^11^ first, each detected SO was cut to 1 second before and after the negative peak of the SO event. For each event-locked 2-second window, Hilbert transforms were used to extract the instantaneous phase of the SO band (0.3–1.5 Hz) and the instantaneous amplitude of the sigma band (12–16 Hz), which is highly correlated with spindle amplitude and density,^58^ and has been previously used to measure SO-spindle coupling.^59^ To avoid filter edge artifacts, the instantaneous phase and amplitude time-series were calculated on the entire signal before running the SO detection. The strength of the coupling between the SO phase and the sigma amplitude was calculated, for each SO, using the normalized direct Phase Amplitude Coupling (ndPAC) method.^16^ The ndPAC is conceptually similar to the traditional mean vector length method,^60^ with two exceptions. First, the amplitude signal is z-scored to help eliminate distortions in the PAC estimate due to direct current components in data. Second, ndPAC includes a statistical thresholding to reject false estimates arising from distortions of non-coupled oscillation powers. As such, and unlike other PAC methods, the ndPAC does not require a permutation-based surrogate normalization. The ndPAC coupling value ranges from 0 (no coupling) to 1 (perfect coupling). Formally, the ndPAC is defined as:

$$ndPAC=\frac{1}{N}\left|\sum_{n=1}^{N}a\left(n\right)e^{i\varphi \left(n\right)}\right|$$

Where a(n) is the normalized (mean removed and variance made unity) amplitude signal and φ(n) is the phase from high- and low-bandpass filtered signals with data length N, respectively. The closed-form statistical threshold is given by:

$$x_{th}=2\times N\times {\left[{\mathrm{erf}}^{-1}\left(1-p\right)\right]}^{2}$$

With p the confidence level, and ${\mathrm{erf}}^{-1}$ the inverse error function.^16^ Every value of coupling exceeding the threshold $x_{th}$ is considered reliable, at the given confidence level. Otherwise, coupling is considered unreliable and values are set to zero. The proportion of SOs that are coupled with the spindle-related sigma band therefore represents a simple metric of the coupling *quantity*. Noteworthy, another approach that has been used to estimate the quantity of SO events that are coupled is to apply an automatic spindle detection on the signal and then find spindles that occur within a certain range of the negative peak of the SO. However, the ndPAC approach has the advantage of being data-driven and as such does not rely on arbitrary thresholds for the spindle detection and events co-occurrence.

A single summary value of coupling strength per participant was obtained by averaging all the valid ndPAC values, that is, all the SO-spindle coupling values that were not rejected by the statistical thresholding. In addition, the proportion of SO that had a valid (= significant) SO-spindle coupling was calculated for each participant. A value of 1 therefore indicates that all the detected SOs have a significant phase-amplitude coupling with the sigma band, whereas a value of zero indicates that none of the detected SOs show a functional coupling with the sigma band. Lastly, the preferred phase (in radians) of the SO at the maximum sigma amplitude within each 2-second window was extracted as a measure of coupling directionality. To this end, the amplitude values were first binned according to 18 phase slices (360 deg/18 bins = 20° each). The preferred phase was then defined as the phase bin for which the distribution of amplitude is maximum.

An outlier removal step was applied which consisted of masking the coupling values with an absolute *Z* score above 4 for either the coupling strength or the coupling quantity (n = 8 in CFS, n = 9 in MESA).

For illustrative purposes, a time-frequency representation of the SO-spindle coupling was calculated using the event-related phase-amplitude coupling (ERPAC) method.^15^ ERPAC is based on a circular-linear correlation that evaluates, across all detected SO for a given night/individual, the instantaneous amplitude at each specific frequency with the sine and cosine of the instantaneous phase. As with a traditional Pearson correlation, values can range between −1 and 1, with higher positive values indicating a strong coupling at that specific event-locked time between the amplitude and phase time series. All coupling analyses were performed in Python using the Tensorpac package.^46^

#### Heart rate variability

Heart rate variability (HRV) across the night was calculated from the ECG channel using non-overlapping windows of 5 minutes. The ECG was first high-pass-filtered at 0.5 Hz using a 5th-order Butterworth filter and the R-peaks were detected and corrected for each 5-min window using the default parameters in the neurokit2 Python toolbox.^61^ Windows with less than 175 NN intervals were excluded. Based on the experimental hypotheses, the analyses were focused on the root-mean-square of successive differences between normal heartbeats (RMSSD) — a widely-used HRV metric that reflects vagally-mediated short-term variability in heart rate.^62^ Of note, although HRV metrics are widely used as a marker of parasympathetic activity, heart rate variability is also impacted by endocrine and reproductive factors, including but not limited to growth hormone,^63^ luteinizing hormone,^64^ and thyroid hormones.^65^ Formally, given a time-series of beat-to-beat interval RR of length N, the RMSSD is defined as:

$$RMSSD=\sqrt{\frac{\sum_{i=1}^{N-1}{\left({RR}_{i}-{RR}_{i+1}\right)}^{2}}{N-1}}$$

The median RMSSD across all 5 minute epochs was calculated to get a single RMSSD value per participant. The resulting values were then log-transformed to reduce skewness, consistent with standard practices.^62^

#### Statistical analyses

A strict inner merge was used to combine the health data (demographics and glucose) with the SO-spindle coupling variables. In other words, only participants with non-missing glucose and coupling data were included in subsequent analyses (n = 647 participants). A more liberal left merge was then used to combine the EEG spectral power data with the main dataframe.

Correlations between dependent variables were calculated using the Pearson correlation coefficient. Partial correlations were performed in Python using the Pingouin package.^47^ All regression analyses were performed using the `lmer` R function.^66^ Models were adjusted for age, gender, race/ethnicity,^67^ BMI, hypertension status, apnea-hypopnea index (AHI), sleep period time (SPT) and sleep efficiency (SE, calculated as total sleep time divided by sleep period time^44^). Since the CFS study includes participants from the same family, multilevel models were used with family ID as a random effect. P-values for the regression models were obtained from two-tailed Wald tests. Marginal effects were calculated using the ‘ggeffect’ R function.^68^

The preprocessing and analysis steps were identical between the CFS main cohort and the MESA replication cohort. One notable exception is that MESA does not include participants from the same families and therefore a standard (non-multilevel) linear regression was used to test associations between predictors of interest and glucose outcomes. A total of 1996 unique MESA participants were remaining after combining the health data and EEG coupling data. There was no participant under the age of 15 years in MESA.

Assessment of the ranking of the sleep predictors was performed by extracting, independently for each sleep predictor, the p value of that predictor in a multilevel regression model adjusted for age, gender, race/ethnicity, BMI, and hypertension status. AHI, SPT and SE were not included as covariates in the model since all three were included, as predictors, in the ranking analysis. The unadjusted p values from all sleep predictors were then log-transformed with base 10 and negated for illustrative purposes.

## Data Availability

• The CFS dataset can be obtained, with the appropriate permissions, and for non-commercial use, at https://sleepdata.org/datasets/cfs. The MESA dataset can be obtained, with the appropriate permissions, through the BioLINCC repository at: https://biolincc.nhlbi.nih.gov/studies/mesa/. In addition to the public access repository, interested investigators may also access the data through the MESA Coordinating Center at the University of Washington. Use of the data via this mechanism is overseen by standard MESA policies and procedures, which assure that participant consent is honored. Additional information can be found at: https://www.mesa-nhlbi.org/. Computer code to reproduce the results of this paper has been deposited at https://github.com/raphaelvallat/vallat2023_coupling_glucose, and will be made publicly available as of the date of publication. • Any additional information required to reanalyze the data reported in this paper is available from the lead contact upon request.

## Supplemental information

Document S1. Figures S1–S4 and Tables S1–S10

Document S2. Article plus supplemental information
