# Supplementary material for: Coordinated human sleeping brainwaves map peripheral body glucose homeostasis
Source: Cell Rep Med. 2023 Jul 7;4(7):101100. doi: 10.1016/j.xcrm.2023.101100 (PMC10394167; doi:10.1016/j.xcrm.2023.101100)

Cell Reports Medicine, Volume *4*

Supplemental information

Coordinated human sleeping brainwaves map peripheral body glucose homeostasis

Raphael Vallat, Vyoma D. Shah, and Matthew P. Walker

Supplementary Tables

Table S1. Demographics of the Cleveland Family Study (CFS; visit 5). Related to STAR Methods.

Variables	Statistics
No. unique participants	647
No. unique families	144
Age (yrs)	44.73 ± 17.39 (range = 15-89)
Sex	359 F / 288 M
BMI (kg/m^2)	33.47 ± 9.17 (range = 17-85)
Race/ethnicity	350 African / 280 Caucasian / 17 Other
Smoking status	324 No / 169 Yes / 154 Missing
Hypertension status	428 No / 211 Yes
Diabetes status	530 No / 115 Yes
Fasting glucose (mg/dl)	100.56 ± 25.65 (range = 66-246)
log(HOMA-IR)	0.99 ± 0.77 (range = -1-4)
log(HOMA-B)	4.81 ± 0.63 (range = 3-8)
Apnea-hypopnea index (AHI)	13.78 ± 20.17 (range = 0-125)
% of participants with AHI >30	13.6%
Sleep efficiency (SE, %)	81.18 ± 12.74 (range = 27-99)
Sleep period time (SPT, min)	459.13 ± 82.01 (range = 52-660)
Total sleep time (TST, min)	369.83 ± 74.26 (range = 40-576)
N1 (% of TST)	5.32 ± 4.72 (range = 0-63)
N2 (% of TST)	58.55 ± 12.41 (range = 21-100)
N3 (% of TST)	17.71 ± 10.99 (range = 0-57)
REM (% of TST)	18.41 ± 7.61 (range = 0-44)
SO density (per min of NREM)	2.50 ± 1.98 (range = 0-11)
Spindles density (per min of NREM)	3.28 ± 1.37 (range = 0-7)
Proportion of SO with significant coupling	87.60 ± 3.35 (range = 72-100)
SO—spindle coupling strength	0.32 ± 0.02 (range = 0.25-0.39)
SO—spindle preferred phase (°)	-12.15 ± 28.32

SO = slow oscillations.

Table S2. Slow oscillation—spindle coupling quantity significantly predicts next-day fasting blood glucose in the CFS dataset. Related to Figure 2.

**Predictors**	**β**	**Standardized β**	**95% CI**	**p**
Proportion of NREM SO with significant coupling	-3.88	-0.12	-6.16 – -1.60	**0.001**
Age	0.01	0.15	0.00 – 0.01	**<0.001**
Male	0.41	0.19	0.26 – 0.57	**<0.001**
Race/ethnicity [Black]	0.15	0.14	-0.04 – 0.34	0.128
Race/ethnicity [Other]	0.18	0.16	-0.34 – 0.69	0.502
BMI	0.03	0.28	0.02 – 0.04	**<0.001**
Hypertension	0.44	0.19	0.25 – 0.62	**<0.001**
Apnea-hypopnea index (AHI)	-0.00	-0.06	-0.01 – 0.00	0.158
Sleep efficiency	-0.01	-0.07	-0.01 – 0.00	0.105
Sleep period time	-0.01	-0.02	-0.07 – 0.05	0.686

The number of participants with complete data included in the multilevel regression analysis was 623. Family ID was set as a random effect (n=144 unique groups). The dependent variable, fasting blood glucose, was transformed using a square root transformation to reduce skewness. The reference category for race/ethnicity was White. Age, sex, BMI, and hypertension were all significant predictors of fasting blood glucose levels. Being a male, older, having a higher BMI, and having hypertension were all associated with higher levels of next-day fasting blood glucose. Race, sleep duration, sleep efficiency, and AHI were not significant predictors of next-day fasting blood glucose.

Table S3. Slow oscillation—spindle coupling strength significantly predicts next-day fasting blood glucose in the CFS dataset. Related to Figure 2.

**Predictors**	**β**	**Standardized β**	**95% CI**	**p**
SO—spindle coupling strength	-4.90	-0.08	-9.04 – -0.77	**0.020**
Age	0.01	0.16	0.00 – 0.02	**<0.001**
Male	0.41	0.18	0.25 – 0.56	**<0.001**
Race/ethnicity [Black]	0.16	0.15	-0.03 – 0.35	0.102
Race/ethnicity [Other]	0.18	0.16	-0.34 – 0.69	0.502
BMI	0.03	0.29	0.03 – 0.04	**<0.001**
Hypertension	0.44	0.19	0.25 – 0.62	**<0.001**
Apnea-hypopnea index (AHI)	-0.00	-0.05	-0.01 – 0.00	0.164
Sleep efficiency	-0.00	-0.05	-0.01 – 0.00	0.198
Sleep period time	-0.01	-0.01	-0.07 – 0.05	0.746

The number of participants with complete data included in the multilevel regression analysis was 623. Family ID was set as a random effect (n=144 unique groups). The dependent variable, fasting blood glucose, was transformed using a square root transformation to reduce skewness. The reference category for race/ethnicity was White. Age, sex, BMI, and hypertension were all significant predictors of fasting glucose levels. Being a male, older, having a higher BMI, and having hypertension were all associated with higher levels of next-day fasting blood glucose. Race, sleep duration, sleep efficiency, and AHI were not significant predictors of next-day fasting blood glucose.

Table S4. Demographics of the MESA sleep study. Related to STAR Methods.

**Variables**	**Statistics**
No. unique participants	1996
Age (yrs)	68.43 ± 9.17 (range = 54-93)
Sex	359 F / 288 M
BMI (kg/m^2)	28.64 ± 5.49 (range = 17-56)
Race/ethnicity	729 Caucasian / 550 African / 478 Hispanic / 239 Asian
Smoking status	930 Never / 912 Former / 142 Current
Gap between PSG and glucose Measures (days)	341 ± 200 (range = 0-1024)
Hypertension status	1130 Yes / 866 No
Diabetes status	1197 Normal / 409 Impaired / 356 Treated / 34 Untreated
Fasting glucose (mg/dl)	100.46 ± 21.81 (range = 62-249)
log(HOMA-IR)	0.99 ± 0.77 (range = -1-4)
log(HOMA-B)	4.81 ± 0.63 (range = 3-8)
Apnea-hypopnea index (AHI)	19.73 ± 18.54 (range = 0-111)
% of participants with AHI >30	21.8%
Sleep efficiency (SE, %)	78.53 ± 13.44 (range = 10-99)
Sleep period time (SPT, min)	462.20 ± 91.48 (range = 94-1084)
Total sleep time (TST, min)	359.89 ± 82.15 (range = 32-601)
N1 (% of TST)	14.18 ± 9.16 (range = 0-79)
N2 (% of TST)	58.00 ± 11.05 (range = 19-100)
N3 (% of TST)	9.98 ± 9.05 (range = 0-51)
REM (% of TST)	17.84 ± 6.92 (range = 0-59)
SO density (per min of NREM)	1.48 ± 1.18 (range = 0-13)
Spindles density (per min of NREM)	2.68 ± 1.43 (range = 0-12)
Proportion of SO with significant coupling	86.34 ± 4.44 (range = 67-100)
SO—spindle coupling strength	0.32 ± 0.02 (range = 0.23-0.41)
SO—spindle preferred phase (°)	-12.15 ± 28.32

SO = slow oscillations.

Table S5. Slow oscillation—spindle coupling quantity significantly predicts next-day fasting blood glucose in the MESA dataset. Related to Figure 3.

**Predictors**	**β**	**Standardized β**	**95% CI**	**p**
Proportion of NREM SO with significant coupling	-1.04	-0.05	-2.00 – -0.08	**0.034**
Age	0.00	0.02	-0.00 – 0.01	0.390
Male	0.22	0.11	0.13 – 0.30	**<0.001**
Race/ethnicity [Black]	0.02	0.02	-0.09 – 0.13	0.688
Race/ethnicity [Asian]	0.37	0.38	0.23 – 0.51	**<0.001**
Race/ethnicity [Hispanic]	0.36	0.37	0.25 – 0.47	**<0.001**
BMI	0.04	0.20	0.03 – 0.04	**<0.001**
Hypertension	0.23	0.12	0.14 – 0.32	**<0.001**
Apnea-hypopnea index (AHI)	0.00	0.03	-0.00 – 0.00	0.201
Sleep efficiency	0.00	0.01	-0.00 – 0.00	0.698
Sleep period time	-0.00	-0.02	-0.00 – 0.00	0.380

The number of participants with complete data included in the multilevel regression analysis was 1966. The dependent variable, fasting blood glucose, was transformed using a square root transformation to reduce skewness. The reference category for race/ethnicity was White. Sex, race BMI, and hypertension were all significant predictors of fasting blood glucose levels. Being male, being Asian, being Hispanic, having a higher BMI, and having hypertension were all associated with higher levels of next-day fasting blood glucose. Age, sleep duration, sleep efficiency, and AHI were not significant predictors of fasting blood glucose.

Table S6. Slow oscillation—spindle coupling strength significantly predicts next-day fasting blood glucose in the MESA dataset. Related to Figure 3.

**Predictors**	**β**	**Standardized β**	**95% CI**	**p**
Proportion of NREM SO with significant coupling	-2.57	-0.06	-4.56 – -0.59	**0.011**
Age	0.00	0.02	-0.00 – 0.01	0.464
Male	0.22	0.11	0.13 – 0.30	**<0.001**
Race/ethnicity [Black]	0.02	0.02	-0.09 – 0.13	0.710
Race/ethnicity [Asian]	0.37	0.38	0.23 – 0.51	**<0.001**
Race/ethnicity [Hispanic]	0.36	0.37	0.25 – 0.47	**<0.001**
BMI	0.03	0.19	0.03 – 0.04	**<0.001**
Hypertension	0.23	0.12	0.14 – 0.32	**<0.001**
Apnea-hypopnea index (AHI)	0.00	0.03	-0.00 – 0.00	0.210
Sleep efficiency	0.00	0.01	-0.00 – 0.00	0.691
Sleep period time	-0.00	-0.02	-0.00 – 0.00	0.387

The number of participants with complete data included in the multilevel regression analysis was 1966. The dependent variable, fasting blood glucose, was transformed using a square root transformation to reduce skewness. The reference category for race/ethnicity was White. Sex, race BMI, and hypertension were all significant predictors of fasting blood glucose levels. Being male, being Asian, being Hispanic, having a higher BMI, and having hypertension were all associated with higher levels of next-day fasting blood glucose. Age, sleep duration, sleep efficiency, and AHI were not significant predictors of fasting blood glucose.

Table S7. Slow oscillation—spindle coupling quantity significantly predicts next-day HOMA-IR in the CFS dataset. Related to Figure 4.

**Predictors**	**β**	**Standardized β**	**95% CI**	**p**
Proportion of NREM SO with significant coupling	-2.20	-0.10	-3.73 – -0.68	**0.005**
Age	-0.0	0.02	-0.00 – 0.00	0.660
Male	0.17	0.11	0.07 – 0.27	**0.001**
Race/ethnicity [Black]	0.14	0.19	0.02 – 0.27	**0.025**
Race/ethnicity [Other]	0.13	0.17	-0.21 – 0.47	0.458
BMI	0.04	0.44	0.03 – 0.04	**<0.001**
Hypertension	0.23	0.14	0.11 – 0.35	**<0.001**
Apnea-hypopnea index (AHI)	0.00	0.01	-0.00 – 0.00	0.826
Sleep efficiency	-0.00	-0.01	-0.01 – 0.00	0.797
Sleep period time	-0.03	-0.05	-0.07 – -0.01	**0.141**

The number of participants with complete data included in the multilevel regression analysis was 626. Family ID was set as a random effect (n=144 unique groups). The dependent variable, HOMA-IR, was log-transformed to reduce skewness. The reference category for race/ethnicity was White.

Table S8. Slow oscillation—spindle coupling strength significantly predicts next-day HOMA-IR in the CFS dataset. Related to Figure 4.

**Predictors**	**β**	**Standardized β**	**95% CI**	**p**
SO—spindle coupling strength	-3.39	-0.08	-6.14 – -0.64	**0.016**
Age	0.00	0.02	-0.00 – 0.00	0.580
Male	0.17	0.11	0.06 – 0.27	**0.002**
Race/ethnicity [Black]	0.15	0.19	0.03 – 0.27	**0.018**
Race/ethnicity [Other]	0.14	0.18	-0.20 – 0.48	0.428The
BMI	0.04	0.44	0.03 – 0.04	**<0.001**
Hypertension	0.23	0.14	0.11 – 0.35	**<0.001**
Apnea-hypopnea index (AHI)	0.00	0.01	-0.00 – 0.00	0.829
Sleep efficiency	0.00	0.00	-0.00 – 0.00	0.973
Sleep period time	-0.03	-0.05	-0.07 – -0.01	**0.170**

The number of participants with complete data included in the multilevel regression analysis was 626. Family ID was set as a random effect (n=144 unique groups). The dependent variable, HOMA-IR, was log-transformed to reduce skewness. The reference category for race/ethnicity was White.

Table S9. Multilevel regression between sleep features and next-day fasting blood glucose in the CFS dataset. Related to Figure 5.

Sleep predictors	Std. beta	n	p
Proportion of coupled SO	-0.109	639	**0.002**
REM alpha	0.097	626	**0.01**
NREM theta	-0.088	647	**0.013**
SO–spindle coupling strength	-0.079	639	**0.026**
REM delta	-0.059	626	0.107
NREM delta (SWA)	0.058	647	0.1
N1	-0.057	647	0.11
REM sigma	0.056	626	0.161
NREM slow delta	0.056	647	0.11
TIB	-0.055	647	0.112
SO density	0.055	647	0.23
Arousal index	-0.049	640	0.197
REM theta	0.049	626	0.171
%N1	-0.047	647	0.2
SME	-0.047	647	0.231
REM fast delta	-0.046	626	0.233
N2 latency	-0.044	647	0.214
NREM alpha	-0.041	647	0.245
REM slow delta	-0.039	626	0.266
SOL	-0.039	647	0.274
WASO	0.037	647	0.333
NREM	-0.034	647	0.354
Spindles frequency	-0.033	647	0.346
N2	-0.033	647	0.349
N3 latency	0.032	617	0.37
TST	-0.032	647	0.392
N3	0.029	647	0.483
N1 latency	-0.028	640	0.438
%N3	0.027	647	0.502
AHI	-0.027	647	0.491
SO frequency	-0.021	647	0.601
NREM beta	-0.02	647	0.56
SO amplitude	-0.02	647	0.664
NREM power	0.019	647	0.58
%REM	0.017	647	0.614
%NREM	-0.017	647	0.616
%N2	-0.015	647	0.696
Spindles power	0.013	647	0.736

REM power	-0.013	626	0.718
NREM fast delta	-0.011	647	0.75
REM	-0.008	647	0.823
REM beta	-0.007	626	0.851
SE	-0.007	647	0.86
REM latency	0.004	626	0.918
Spindles density	-0.004	647	0.921
NREM sigma	-0.003	647	0.927
SPT	0.001	647	0.988

All regressions were adjusted for age, sex, BMI, race/ethnicity, hypertension and family ID. Regressions are sorted in descending order of significance. NREM refers to N2 + N3 sleep (N1 excluded). Sleep features that significantly predict *higher* levels of fasting glucose = worse outcome) are highlighted in red. A total of 47 sleep parameters were included in the correlation analysis. Two-sided p-values were not corrected for multiple comparisons. The spectral frequency bands are: slow delta (0.5-1.25 Hz), fast delta (1.25-4 Hz), delta (0.5-4 Hz), theta (4-8 Hz), alpha (8-12 Hz), sigma (12-16 Hz), beta (16-30 Hz), and total power (in microvolts-squared, 0.5-30 Hz). AHI = Apnea–hypopnea index, SE = sleep efficiency, SO = slow oscillations, SME = sleep maintenance efficiency, SOL = sleep onset latency, SPT = sleep period time, TST = total sleep time, WASO = wake after sleep onset.

Table S10. Multilevel regression between sleep features and next-day insulin resistance (HOMA-IR) in the CFS dataset. Related to Figure 5.

Sleep predictors	Std. beta	n	p
SO density	0.11	647	**0.012**
Proportion of coupled SO	-0.096	639	**0.004**
SO–spindle coupling strength	-0.084	639	**0.014**
REM alpha	0.083	626	**0.022**
NREM fast delta	-0.081	647	**0.017**
NREM slow delta	0.079	647	**0.019**
Spindles density	-0.072	647	**0.039**
NREM theta	-0.072	647	**0.035**
REM theta	0.068	626	**0.047**
SO amplitude	0.067	647	0.124
REM delta	-0.059	626	0.099
%N1	-0.059	647	0.095
N1	-0.058	647	0.095
%N3	0.056	647	0.145
SPT	-0.052	647	0.123
REM	-0.05	647	0.147
REM fast delta	-0.05	626	0.178
TST	-0.049	647	0.182
SOL	0.047	647	0.175
N3	0.045	647	0.256
SO frequency	-0.041	647	0.297
NREM delta (SWA)	0.039	647	0.252
NREM power	0.039	647	0.246
N3 latency	0.039	617	0.274
REM slow delta	-0.037	626	0.285
N1 latency	0.036	640	0.297
N2	-0.036	647	0.292
REM latency	0.035	626	0.314
SE	-0.033	647	0.356
NREM	-0.028	647	0.436
N2 latency	0.027	647	0.427
NREM beta	-0.022	647	0.511
AHI	0.022	647	0.561
Spindles frequency	-0.022	647	0.526
SME	0.021	647	0.571
WASO	-0.02	647	0.583
REM sigma	0.02	626	0.605
REM power	0.017	626	0.615

TIB	-0.017	647	0.616
NREM alpha	-0.014	647	0.677
%NREM	0.013	647	0.703
%REM	-0.013	647	0.706
Spindles power	0.012	647	0.738
%N2	-0.011	647	0.752
REM beta	-0.008	626	0.812
NREM sigma	0.004	647	0.9
Arousal index	-0.001	640	0.978

All regressions were adjusted for age, sex, BMI, race/ethnicity, hypertension and family ID. Regressions are sorted in descending order of significance. NREM refers to N2 + N3 sleep (N1 excluded). Sleep features that significantly predict *higher* HOMA-IR values (= worse outcome) are highlighted in red. A total of 47 sleep parameters were included in the correlation analysis. Two-sided p-values were not corrected for multiple comparisons. The spectral frequency bands are: slow delta (0.5-1.25 Hz), fast delta (1.25-4 Hz), delta (0.5-4 Hz), theta (4-8 Hz), alpha (8-12 Hz), sigma (12-16 Hz), beta (16-30 Hz), and total power (in microvolts-squared, 0.5-30 Hz). AHI = Apnea–hypopnea index, SE = sleep efficiency, SO = slow oscillations, SME = sleep maintenance efficiency, SOL = sleep onset latency, SPT = sleep period time, TST = total sleep time, WASO = wake after sleep onset.

Supplementary Figures

Figure S1. Assessment of insulin resistance (IR) and pancreatic beta cells function (B) using the standardized homeostasis assessment model (HOMA). A) HOMA-IR is positively correlated with fasting glucose. B) HOMA-B is negatively correlated with fasting glucose. Related to STAR Methods.

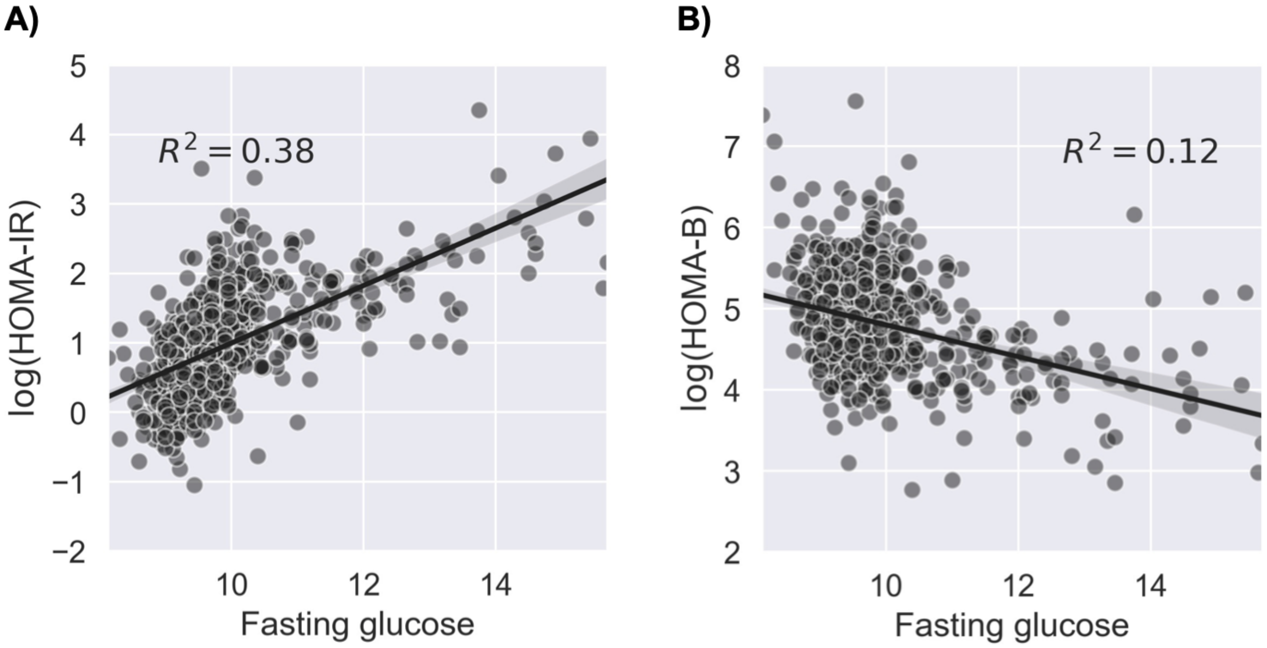

Figure S2. Mediation analysis demonstrated that the link between SO-spindle coupling and improved next-day fasting glucose is, in part, explained by increased heart rate variability, in the MESA dataset. A) A significant association between the proportion of SO-spindle coupling and increased heart rate variability (HRV), which in turn predicted lower (improved) fasting glucose values. B) A significant association between the strength of SO-spindle coupling and increased HRV, which in turn predicted lower (improved) fasting glucose values. Related to Figure 3.

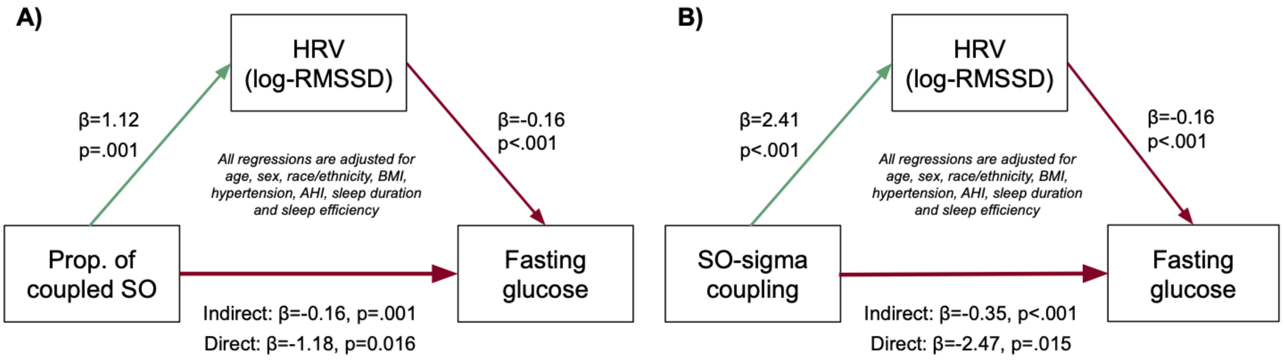

Figure S3. Mediation analysis demonstrated that the link between SO-spindle coupling and improved next-day fasting glucose is not, in part, explained by increased heart rate variability, in the CFS dataset. A) A significant association between the proportion of SO-spindle coupling and lower (improved) fasting glucose values is not mediated by HRV. B) A significant association between the strength of SO-spindle coupling and lower (improved) fasting glucose values is not mediated by HRV. Related to Figure 2.

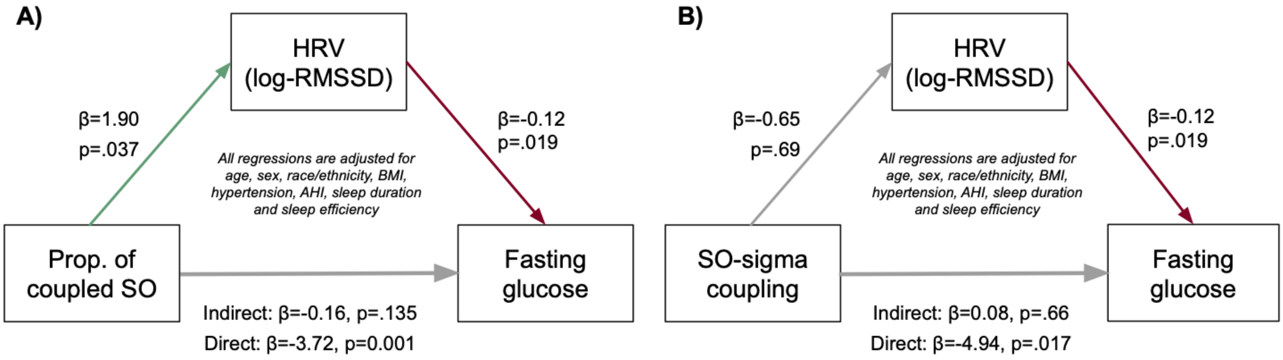

Figure S4. Mediation analysis demonstrated that the link between SO-spindle coupling and next-day insulin resistance is modestly explained by increased heart rate variability, in the CFS dataset. A) A trending significant association between the proportion of SO-spindle coupling increased heart rate variability (HRV), which in turn predicted lower (better) insulin resistance values. B) A significant association between the strength of SO-spindle coupling and lower (better) insulin resistance values is not mediated by HRV. Related to Figure 2.

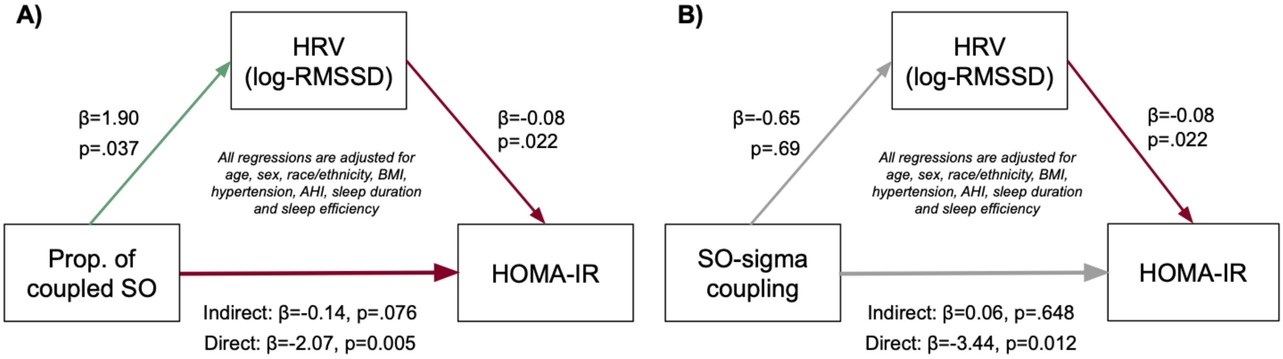

Supplement: Document S1. Figures S1–S4 and Tables S1–S10 [file mmc1.pdf]
